# Local Fungi Promote Plant Growth by Positively Affecting Rhizosphere Metabolites to Drive Beneficial Microbial Assembly

**DOI:** 10.3390/microorganisms13081752

**Published:** 2025-07-26

**Authors:** Deyu Dong, Zhanling Xie, Jing Guo, Bao Wang, Qingqing Peng, Jiabao Yang, Baojie Deng, Yuan Gao, Yuting Guo, Xueting Fa, Jianing Yu

**Affiliations:** College of Ecological and Environment Engineering, Qinghai University, Xining 810016, China; dongdeyu2020@163.com (D.D.);

**Keywords:** local fungi, rhizosphere metabolites, beneficial microorganisms, promoting plant growth, ecological restoration

## Abstract

Ecological restoration in the cold and high-altitude mining areas of the Qinghai–Tibet Plateau is faced with dual challenges of extreme environments and insufficient microbial adaptability. This study aimed to screen local microbial resources with both extreme environmental adaptability and plant-growth-promoting functions. Local fungi (DK; F18-3) and commercially available bacteria (B0) were used as materials to explore their regulatory mechanisms for plant growth, soil physicochemical factors, microbial communities, and metabolic profiles in the field. Compared to bacterial treatments, local fungi treatments exhibited stronger ecological restoration efficacy. In addition, the DK and F18-3 strains, respectively, increased shoot and root biomass by 23.43% and 195.58% and significantly enhanced soil nutrient content and enzyme activity. Microbiome analysis further implied that, compared with the CK, DK treatment could significantly improve the α-diversity of fungi in the rhizosphere soil (the Shannon index increased by 14.27%) and increased the amount of unique bacterial genera in the rhizosphere soil of plants, totaling fourteen genera. Meanwhile, this aggregated the most biomarkers and beneficial microorganisms and strengthened the interactions among beneficial microorganisms. After DK treatment, twenty of the positively accumulated differential metabolites (DMs) in the plant rhizosphere were highly positively associated with six plant traits such as shoot length and root length, as well as beneficial microorganisms (e.g., *Apodus* and *Pseudogymnoascus*), but two DMs were highly negatively related to plant pathogenic fungi (including *Cistella* and *Alternaria*). Specifically, DK mainly inhibited the growth of pathogenic fungi through regulating the accumulation of D-(+)-Malic acid and Gamma-Aminobutyric acid (*Cistella* and *Alternaria* decreased by 84.20% and 58.53%, respectively). In contrast, the F18-3 strain mainly exerted its antibacterial effect by enriching *Acidovorax* genus microorganisms. This study verified the core role of local fungi in the restoration of mining areas in the Qinghai–Tibet Plateau and provided a new direction for the development of microbial agents for ecological restoration in the Qinghai–Tibet Plateau.

## 1. Introduction

Mining is known to cause extremely severe damage to the vegetation layer and mainly results in the covering and erosion of topsoil, the loss of organic matter and nutrients, and the regeneration of vegetation without physical protection and that lacks the supply of nutrients necessary for its growth [1,2,3]. Heavy metal accumulation occurs after coal mining and produces toxic substances such as sulfides and polycyclic aromatic hydrocarbons, which inhibit plant root development and harm plant and soil organisms [4]. The accumulation of these substances leads to a reduction in beneficial soil microorganisms (including bacteria, fungi, actinomycetes, etc.) and soil organisms, such as arthropods and nematodes, and damage to ecological functions [5,6]. Hence, it is essential to develop a technical approach that can effectively alleviate damage to plants in mining areas and increase the beneficial microorganisms in the soil for ecological restoration after mining.

In recent years, beneficial microorganisms (microbial agents) have shown great potential in the ecological restoration of mining areas, promoting plant growth, enhancing plant stress resistance and soil microorganism–plant interactions, providing new solutions for mine area restoration [7,8,9]. Among them, due to their long-term adaptation to extreme environments, local fungi exhibit unique advantages in mine restoration. Relevant research has accumulated rich results in laboratory and small-scale field trials. For instance, *Aspergillus* isolated from metal mines can lower the bioavailability of soil metals through biosorption and transformation, and it can tolerate Cd (0–100 ppm) and Pb (0–400 ppm) [10]. *Penicillium janthinellum* ZZ-2 isolated from heavy-metal-contaminated soil has a greater capacity to adsorb Cd^2+^ and produces indole acetic acid (IAA), promoting plant growth in the presence of Cd [11]. In terms of plant growth promotion, the role of local fungi is particularly prominent. Endophytic fungi isolated from mining areas (such as dark septate endophytes, DSE) can promote plant growth by regulating the balance of plant hormones, dissolving phosphorus, and synthesizing iron carriers while also inducing plants’ adaptability to metal pollution [12].

However, current explorations on microbial agents are still mainly at the laboratory stage, and research on the application of microbial agents in promoting plant growth at extremely high altitudes (above 4000 m) is relatively scarce. Some people have utilized plant–microbe symbiosis to restore vegetation. For example, arbuscular mycorrhizal fungi (AMF) were used to expand the root system of plants, increase the absorption area of water and nutrients, and enhance plants’ stress resistance [13,14,15]. Plant-growth-promoting microbes were adopted to form symbiosis with plants to improve their tolerance to heavy metals [16,17]. Zhao et al. utilized a microbial compound agent—buckwheat—to restore coal mining areas; after 150 days of restoration, soil fertility was significantly enhanced, while heavy metal accumulation was significantly reduced [18]. Huang et al. verified that inoculating AM fungi was beneficial for improving the ecological restoration of abandoned ionic rare earth mining areas, neutralizing acidic soil, increasing nutrient content, and promoting plant growth [19]. Some researchers restored coal mine land by planting ryegrass, adding sludge and inoculating AMF. After six consecutive years of field experiments, the texture of the coal mine land was significantly improved [20].

However, it should be noted that most of the microbial agents currently available on the market have been developed for agricultural environments in temperate or tropical regions, and their strains and application conditions are significantly different from the cold environment of the Qinghai–Tibet Plateau. In contrast, local fungi isolated from the Qinghai–Tibet Plateau have adapted to the extreme environmental conditions through long-term natural selection and evolution and possess stronger environmental adaptability. It has been found that two strains of fungi isolated from the high-altitude regions of the Qinghai–Tibet Plateau (*Trichoderma* DK and *Floccularia luteovirens* F18-3) can adapt to low-temperature environments and degrade polycyclic aromatic hydrocarbons (PAHs) and other aromatic compounds. At the same time, they facilitate plant growth [21]. Therefore, it was hypothesized that the specific metabolites induced by these local plant-growth-promoting fungi might regulate the microbiota related to plant growth and promote the growth of plants in the extremely high-altitude coal mining areas of the Qinghai–Tibet Plateau.

Currently, there is little work on the use of microbial agents to restore vegetation in mining areas, and there are few reports on the development of microbial agents specifically for the unique geographic environment of the Qinghai–Tibet Plateau. The present work aimed to ① study the regulatory effects of local fungi in the Qinghai–Tibet Plateau on the growth of forage, the structure of rhizosphere microbial communities, and the composition of metabolites in extremely high-altitude (above 4000 m) coal mining areas and explore their application potential in the restoration of vegetation in those areas; ② screen out high-quality plant-growth-promoting strains adapted to extreme cold and high-altitude environments, providing theoretical basis and strain resources for the development of specialized microbial preparations for extremely high-altitude mining areas in the Qinghai–Tibet Plateau; and ③ reveal the mechanism by which local fungi promote the growth of forage in extremely high-altitude coal mining areas through regulating the interaction between rhizosphere metabolites and microorganisms, providing new ideas and methods for the restoration and protection of ecosystems in extremely cold and high-altitude mining areas.

## 2. Materials and Methods

### 2.1. Test Strains

The following three strains were selected as experimental materials in this experiment (Table S1): (1) *Trichoderma* sp. DK (CGMCC No. 40932, accession no. (ITS) KR995112.1), isolated from the root of *Kobresia humilis* on the Qinghai–Tibet Plateau. Isolation method: Fresh roots were collected and surface sterilized with 75% ethanol for 30 s, followed by 5.25% sodium hypochlorite solution for 10 min, and then rinsed five times with sterile water. The roots were then cut into 0.1–0.3 cm fragments and inoculated onto PDA medium (Potato Dextrose Agar, 200 g/L potato, 20 g/L glucose, 17 g/L agar) and incubated in the dark at 20 °C for 5–7 days. Marginal mycelia were picked for single colony purification (repeated purification of the strain multiple times), and the species was confirmed through morphological observation and ITS sequence comparison. (2) *Floccularia luteovirens* F18-3 (CGMCC No. 40991, accession no (ITS). MK955328, accession no. (LSU) MT950058), isolated from the fruiting body of wild edible mushroom *Floccularia luteovirens* on the Qinghai–Tibet Plateau. Isolation method: The surface of the fruiting bodies was wiped with 75% ethanol for disinfection, and the flesh tissue was aseptically removed and inoculated into VB liquid medium (exogenous mycorrhizal culture medium, 200 g/L potato, 20 g/L glucose, 1.5 g/L MgSO_4_•7H_2_O, 3.0 g/L KH_2_PO_4_, 0.01 g/L vitamin B1, and 17 g/L agar) and incubated at 20 °C for 10–15 days. The strain was repeatedly purified to obtain a single colony, and the identity was verified through ITS sequence (primers ITS1/ITS4) and LSU gene (primers LSU F/LSU R) sequencing, confirming 99.7% homology with *Floccularia luteovirens* [22]. B0 (*Bacillus subtilis*), as a widely used biocontrol strain in agriculture, was used as the control strain, and its fermentation broth was a commercially purchased product.

### 2.2. Preparation of Strain Fermentation Broth

*Trichoderma* sp. DK was cultivated in a 5 L Erlenmeyer flask containing 4 L PDA liquid medium at 25 °C for 7 days. *Floccularia luteovirens* F18-3 was cultivated in the same 5 L flask with 4 L VB liquid medium at 25 °C for 30 days [21]. An amount of 400 mL of the strain seed culture was inoculated into an Erlenmeyer flask containing 4 L of liquid medium. The seed culture was required to achieve a mycelial dry weight of 0.46 g/100 mL. During the cultivation process, mycelial growth was monitored. After that, the fermentation broth was collected for subsequent experiments.

### 2.3. Test Plants

Four perennial forage cultivars were selected as test plant materials (Table S2), namely *Elymus breviaristatus* cv. Tongde, *Festuca sinensis* Keng cv. Qinghai, *Poa crymophila* Keng cv. Qinghai, and *Poa pratensis* L. cv. Qinghai. All the seeds of these forages were provided by the Qinghai Provincial Grassland Station.

### 2.4. Substrate and Mixing Materials

Sheep manure was used as the base fertilizer (25 m^3^/667 m^2^). The fermentation broth of the DK strain, F18-3 strain, and B0 strain, as well as control water, were, respectively, thoroughly mixed with the base fertilizer at a ratio of 1 L:1 m^3^. Each treatment only contained one strain’s fermentation broth or the control water, and the four treatments were independent of each other. Then, the mixtures obtained from each treatment were evenly applied to the soil surface and thoroughly mixed with the soil through mechanical tillage (tillage depth of 20 cm) to ensure uniform mixing of the mixtures and soil.

### 2.5. Overview of Experimental Field and Experimental Design

#### 2.5.1. Overview of Experimental Area

The research area was situated in Wuli Town, Tianjun County, within the Haixi Mongolian and Tibetan Autonomous Prefecture of Qinghai Province (38°09′41′′ N, 99°09′49′′ E, 4126 m). It features a continental plateau subarctic humid climate, with an average annual temperature of −5.3 °C and mean temperatures of −17.2 °C in January and 15.6 °C in July. Annual precipitation ranges from 282 to 774 mm. There is no frost-free period throughout the year. The annual sunshine duration is 3004 h, and solar radiation is 160 kcal/cm^2^ [23].

#### 2.5.2. Design of Experimental Plots and Sowing

A completely randomized design with three experimental plots was adopted for the four treatments: (1) DK: *Trichoderma* sp. DK fermentation broth treatment; (2) F18-3: *Floccularia luteovirens* F18-3 fermentation broth treatment; (3) B0: *Bacillus subtilis* B0 fermentation broth treatment; and (4) CK: water control. Three experimental plots were set up for each treatment, with each plot measuring 1120 m^2^ (40 m × 28 m), and with both the plot spacing and treatment spacing set at 1 m (Figure S1). Sowing was carried out on 14 June 2022 using a seeder. The seed ratio was *Elymus breviaristatus* cv. Tongde: *Festuca sinensis* Keng cv. Qinghai: *Poa crymophila* Keng cv. Qinghai: *Poa pratensis* L. cv. Qinghai = 1:1:1:3, with a sowing rate of 6 kg/667 m^2^.

### 2.6. Field Management

After sowing, the surface was covered with non-woven fabric and fenced for enclosure. No artificial intervention was carried out throughout the growth period. The initial soil conditions are presented in Table S3.

### 2.7. Sample Collection and Processing

#### 2.7.1. Plant Sample Collection

On 16 August 2023 (the peak growth period of forage), three representative plots (25 m^2^/plot, with a 5 m distance between plots) with uniform growth were selected in each experimental area. In total, 36 intact plants were collected from each plot using a 9-point sampling method. The samples were immediately stored in sterile self-sealing bags, stored in ice boxes at 4 °C, and then quickly placed in the laboratory [24]. The mixed samples from the three plots were classified into two equal parts: one was utilized to measure the growth indicators of forage, and the other one was used to measure the physiological and biochemical properties of the forage leaves and the microbial community structure of the root. For root sample pretreatment (microbiome structure analysis), the roots were rinsed with tap water to remove adhering soil, surface sterilized with 75% ethanol (*v*/*v*) for 10 min, and rinsed four times with sterile distilled water, and the surface moisture was absorbed with sterile filter paper. The samples were then aliquoted into cryotubes, flash-frozen in liquid nitrogen, and stored at −80 °C for ultra-low-temperature preservation.

#### 2.7.2. Rhizosphere Soil Collection and Processing

Rhizosphere soil was collected using the standard rhizosphere soil collection method [25]. Specifically, the collected forage roots were gently shaken to remove loose adhering soil, and the tightly adhering soil on the root surface was scraped off with a sterile brush and passed through a 2 mm sieve to remove the impurities. The rhizosphere soil was classified into three parts: the first part (fresh sample) was immediately used for high-throughput sequencing of microorganisms and metabolomics analysis; the second part (fresh sample) was stored at 4 °C, and soil enzyme activity was determined within 24 h; and the third part was air-dried, ground, and sieved to identify the soil physical and chemical properties.

### 2.8. Forage Phenotype and Biochemical Measurement

Shoot length (from the base of the shoot to the top bud), root length (from the tip of the main root to the root collar), and stem diameter (at the base of the shoot) were measured with the help of a ruler and a vernier caliper. Forage biomass was measured using an electronic balance, and the number of effective tillers per plant (length ≥ 2 cm) was manually counted. A total of 20–26 plants were measured for each replicate. Leaf physiological parameters were determined using standardized kits (Nanjing Jiancheng Bioengineering Institute, Nanjing, China): chlorophyll, proline (PRO), total protein, malondialdehyde (MDA), and soluble sugar (SSu) content [26].

### 2.9. Rhizosphere Soil Physical and Chemical Properties and Enzyme Activity Measurement

Soil nutrient indicators in the rhizosphere were determined according to standard chemical analysis methods: soil organic carbon (SOC), total nitrogen (TN), total phosphorus (TP), available potassium (AP), available nitrogen (AN), and available potassium (AK) [27]. The activities of amylase (S-AL), glucosidase (S-GC), polyphenol oxidase (S-PPO), and cellulase (S-CL) in the rhizosphere soil were measured using commercial enzyme kits (Solarbio, Beijing, China) [28,29].

### 2.10. Root and Rhizosphere Soil Microbiome Analysis

Microbial DNA was extracted via employing the HiPure Soil DNA kit (Magen, Guangzhou, China). For fungal analysis, the ITS1 region underwent amplification [30], whereas bacterial profiling targeted the V5–V7 region of 16s rRNA according to a previous study [31]. Amplification product integrity was verified via 2% agarose gel electrophoresis. Subsequent purification utilized AMPure XP Beads (Beckman, CA, USA), followed by quantification by means of Qubit 3.0.

Raw Illumina sequencing data underwent filtration via FASTP (version 0.18.0) [32], yielding clean reads for downstream processing. These clean reads were subsequently merged into tags employing FLASH (version 1.2.11) [33]. Consistent with established filtering criteria, low-quality tags were discarded [34]. Utilizing the UPARSE (version 9.2.64) pipeline [35], clean tags were clustered into operational taxonomic units (OTUs) defined by ≥97% sequence similarity. Within each OTU, the most abundant tag sequence was designated as its representative sequence. Taxonomic assignment of representative OTU sequences was performed using the RDP classifier (version 2.2), leveraging the SILVA database (version 138.1) for bacterial classification and the UNITE database (version 8.3) for fungal identification, implemented through a naive Bayes model [36].

Krona (version 2.6) visualized taxonomic abundance distributions [37]. Community composition was depicted via stacked bar plots generated with R’s ggplot2 package (version 2.2.1) [38]. Shared and unique OTUs/genera across groups were identified through Venn analysis using either the VennDiagram (version 1.6.16) or UpSetR package (version 1.3.3) in R [39,40]. Alpha diversity indices (Shannon and Sobs) were computed in QIIME (version 1.9.1) [41]. Rarefaction curves and rank–abundance distributions based on Shannon indices were plotted employing ggplot2 [38]. Inter-group Shannon index comparisons employed the Kruskal–Wallis H test within R’s Vegan package (version 2.5.3) [42]. To evaluate microbial compositional differences among treatments, multivariate approaches including principal coordinate analysis (PCoA) based on OTU-level Bray–Curtis distances were used in Vegan, with results visualized using ggplot2 [38,42]. Permutational multivariate analysis (adonis) and analysis of similarities (anosim) were executed via the Vegan package [42]. Biomarker discovery for respective groups was carried out by LEfSe (version 1.0) [43]. KEGG pathway prediction was performed via to infer FAPROTAX Bacterial functional potential [44]. Fungal functional guild assignment utilized FUNGuild (version 1.0) [45].

### 2.11. Determination of Rhizosphere Soil Metabolites

Rhizosphere soil samples (0.5 g) from DK, F18-3, B0, and CK treatments were used for analysis via LC-MS/MS. Raw data files were processed in XCMS for peak alignment, detection, and metabolite quantification. Peak intensities underwent normalization against the first sample’s total spectral intensity. Metabolite identification involved matching within a 10 ppm mass deviation window against high-confidence spectral libraries, yielding accurate qualitative and relative quantitative results [46].

Metabolome data normalization utilized z-scores prior to hierarchical clustering via R’s pheatmap package (version 1.0.12). Inter-group comparisons implemented OPLS-DA (Orthogonal Partial Least Squares Discriminant Analysis) using the ropls package (version 1.34.0) [47], with model validity confirmed through cross-validation and permutation tests. Significantly differential metabolites (DMs) were screened by integrating OPLS-DA VIP values (VIP ≥ 1) and univariate *t*-test *p*-values (*p* < 0.05). Annotated DMs underwent KEGG pathway mapping for functional enrichment analysis.

### 2.12. Data Analysis

SPSS 26.0 was used to perform Student’s *t*-test, joint hypotheses test (F-test), and one-way ANOVA to calculate significant differences in forage growth, leaf physiology and biochemistry, soil variable, and microbial taxon composition of the two groups. The correlation coefficients among microorganisms were obtained using the ggcor package (version 0.9.8.1) in R language, and the microbial interaction network was visualized using Cytoscape (version 3.7.0) [48]. The Procrustes analysis was performed on the TuTu Cloud platform (a free online data analysis website) [49]. Partial Mantel tests were utilized to examine correlations between forage growth, soil properties, microbial community structure, and metabolites [50].

## 3. Results

### 3.1. Effects of Strain Treatments on Plant Growth and Physicochemical Factors of Rhizosphere Soil

**Figure 1:**
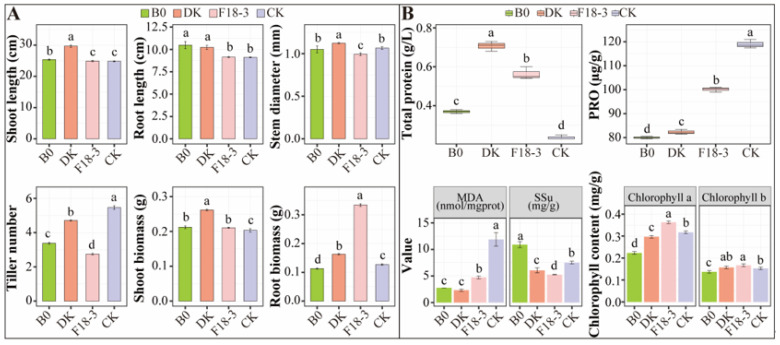
Effects of strain treatment on plant growth and leaf biochemical contents. (**A**) Plant growth, including shoot length, root length, stem diameter, tiller number, shoot biomass, and root biomass. (**B**) Leaf biochemical contents, including total protein, PRO, MDA, SSu. and chlorophyll content. Different lowercase letters above column represent significant differences (*p* < 0.05), *n* = 3.

The local fungus DK had the most significant effect on promoting the growth of plants, while local fungus F18-3 promoted the accumulation of chlorophyll and underground biomass in plants (Figure 1). Specifically, compared with the CK, the DK treatment significantly (*p* < 0.05) increased the shoot length (19.54%), stem diameter (5.31%), shoot biomass (28.69%), root length (12.33%), root biomass (28.35%), and total protein (198.59%) of plants but significantly decreased PRO (30.81%), MDA (80.49%), SSu (19.36%), and chlorophyll a (6.32%). The F18-3 treatment significantly increased the shoot biomass (3.61%), root biomass (162.99%), total protein (138.03%), chlorophyll a (14.74%), and chlorophyll b (8.70%) of plants but significantly decreased the stem diameter (6.88%), tiller number (49.70%), PRO (15.83%), MDA (60.20%), and SSu (30.23%). The control strain B0 treatment significantly increased the shoot length (2.27%), root length (15.0%), stem biomass (4.26%), total protein (56.34%), and SSu (45.32%) of plants but significantly decreased the tiller number (38.17%), root biomass (11.02%), PRO (32.77%), MDA (76.71%), chlorophyll a (29.47%), and chlorophyll b (10.87%).

Strain treatments (DK, F18-3, and B0) had significant differentiation effects on physicochemical factors of rhizosphere soil (Figure S2). Specifically, the B0 treatment significantly reduced the nutrient content and enzyme activity, while compared with B0, the DK and F18-3 treatments significantly increased the nutrient content and enzyme activity (S-AL, S-GC, and S-CL).

### 3.2. Strain Treatments Differentially Shapes the Endophytic and Rhizosphere Microbial Community Structures of Plants

#### 3.2.1. Overview of Microbial Community Composition

A total of 3,877,522 high-quality reads (2,664,756 for fungi and 1,212,766 for bacteria) were obtained through high-throughput sequencing, which were clustered into 10,378 OTUs (1790 for fungi and 8588 for bacteria). Fungal OTUs were divided into 10 phyla, 31 classes, 79 orders, 162 families, and 281 genera (Table S4), while bacterial OTUs were divided into 30 phyla, 90 classes, 204 orders, 311 families, and 445 genera (Table S5). The saturated rarefaction curves (Figure S3A,B) and rank abundance curves (Figure S3C,D) of the fungal and bacterial communities indicated that the sequencing depth of the samples was sufficient to reflect the microbial communities in each sample.

#### 3.2.2. Differential Characteristics of Microbial Community Structure

Venn diagrams were constructed at the OTU level to represent the numbers of shared and unique microorganisms among all plant niches, and the top 10 dominant microbial community was constructed at the phylum and order levels. Compared to the CK group (Figure S4), DK and F18-3 treatments improve the amount of unique fungal and bacterial OTUs in the rhizosphere soil but reduce the amount of unique fungal and bacterial OTUs in the plant roots.

**Figure 2:**
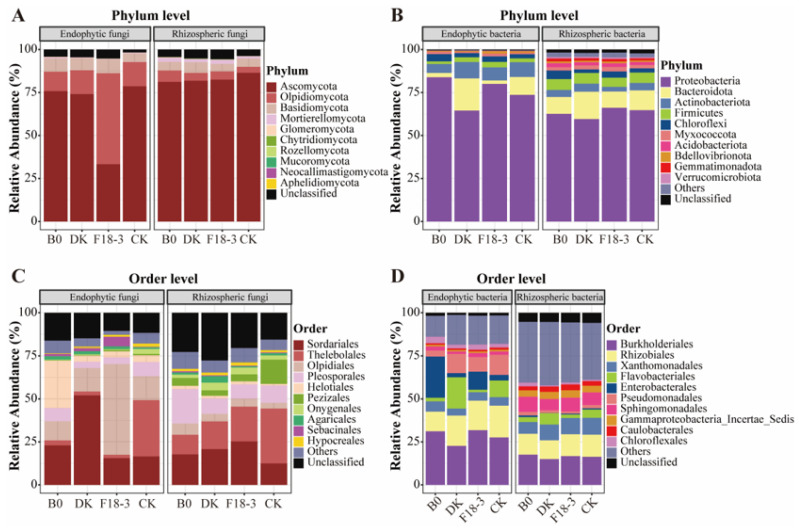
Strain treatment drives assembly changes in endophytic and rhizosphere microbial community structures of plants. Composition of fungi (**A**) and bacteria (**B**) at phylum level and composition of fungi (**C**) and bacteria (**D**) at order level. Top 10 relative abundances of fungal and bacterial compositions.

As can be observed from Figure 2A,B, the dominant fungal communities were mainly Ascomycota (33.33–86.50%), Olpidiomycota (3.42–52.87%), and Basidiomycota (4.48–8.14%), while the bacterial communities were dominated by Proteobacteria (59.62–83.92%), Bacteroidota (1.99–18.67%), Actinobacteriota (2.71–9.52%), and Firmicutes (1.54–6.29%). At the order level (Figure 2C,D), the dominant fungal groups were Sordariales (12.54–52.08%), Thelebolales (1.87–32.65%), Olpidiales (3.42–52.87%), and Pleosporales (3.63–20.04%), while the dominant bacterial groups were Burkholderiales (15.05–31.87%), Rhizobiales (10.90–18.47%), Xanthomonadales (4.07–9.83%), and Flavobacteriales (1.46–18.21%).

**Figure 3:**
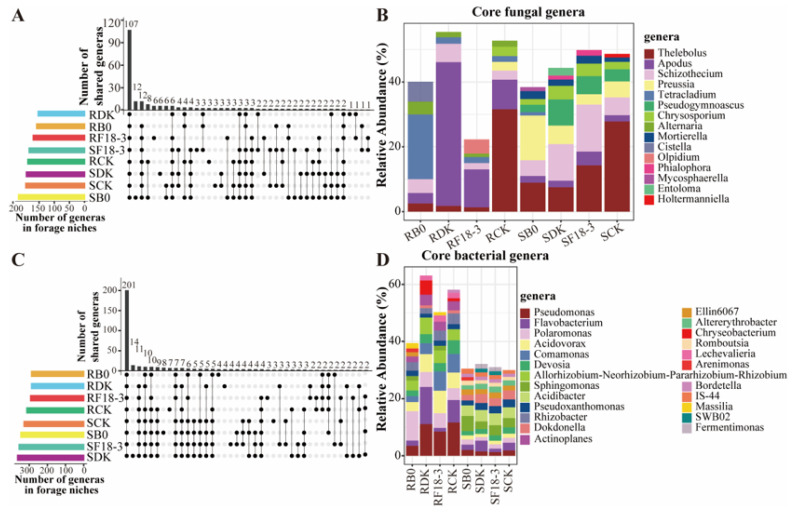
Response analysis of the composition and abundance of microbial communities at the genus level in plant niches to strain treatments. The number of fungal genera (**A**) and bacterial genera (**C**) in plant niches under different strain treatments. The histogram in the lower left corner indicates the number of microbial genera in each plant niche, and the bar chart on the right shows the number of various microbial sets intersecting in plant niches. The single points below represent specific genera in the microbial sets, and the connections between some points indicate the number of shared genera. Stacked bar charts of the relative abundance of shared fungal genera (**B**) and bacterial genera (**D**). Relative abundances > 1%.

Next, at the genus level, the microbial composition of plants under different treatments was explored. A total of 281 fungal genera were identified in the plant roots and rhizosphere soil, among which 107 genera were shared by all groups, and RB0, RDK, RF18-3, RCK, SB0, SDK, SF18-3, and SCK had 0, 1, 6, 3, 12, 6, 3, and 3 unique genera, respectively (Figure 3A; Table S6). The endophytic fungal genera *Apodus* (44.35%) and *Schizothecium* (5.69%), as well as the rhizosphere fungi *Pseudogymnoascus* (8.07%) and *Chrysosporium* (4.19%), were more abundant in the plants treated with DK. The endophytic fungus *Olpidium* (4.37%) and the rhizosphere fungi *Schizothecium* (14.49%) and *Apodus* (4.26%) were more prevalent in plants treated with F18-3. The endophytic fungi *Tetracladium* (19.96%) and *Cistella* (6.11%), as well as the rhizosphere fungus *Preussia* (13.30%), were more abundant in plants treated with B0. However, *Thelebolus* was the most abundant endophytic and rhizosphere fungus in the plant of the CK group, accounting for 31.60% and 27.83%, respectively (Figure 3B).

For bacterial genera, a total of 201 genera were shared by all groups, with 4, 4, 3, 7, 8, 14, 4, and 3 unique genera in RB0, RDK, RF18-3, RCK, SB0, SDK, SF18-3, and SCK, respectively (Figure 3C; Table S6). The plants treated with DK had more abundant endophytic bacteria, including *Flavobacterium* (12.99%) and *Allorhizobium*-*Neorhizobium*-*Pararhizobium*-*Rhizobium* (5.80%), and rhizosphere bacterium *Flavobacterium* (3.80%), while plants treated with F18-3 had a higher abundance of endophytic bacteria, including *Acidovorax* (7.88%) and *Comamonas* (6.77%), and rhizosphere bacterium *Acidibacter* (4.17%). The plants treated with B0 had a higher abundance of endophytic bacteria, namely *Polaromonas* (10.26%), and rhizosphere bacteria, namely *Sphingomonas* (5.30%), while *Pseudomonas* (11.65%) was found to be the most abundant endophytic bacterium in the plants of the CK group (Figure 3D).

Most of the microbial groups with relatively high abundance in plant roots could also be found in the rhizosphere soil. In summary, these microbial taxa colonized the plants from underground.

### 3.3. Strain Treatments Drive the Assembly of Endophytic and Rhizosphere Microbial Communities in Plants

**Figure 4:**
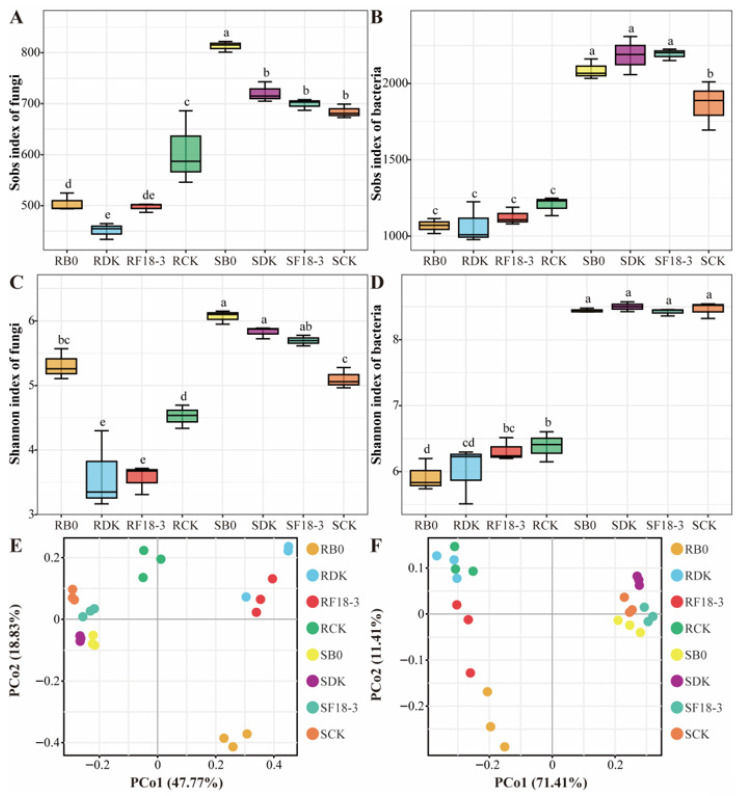
The effects of different strain treatments on the alpha and beta diversity of microorganisms in plant niches. The box plots of alpha diversity show the Sobs index (**A**) and Shannon index (**C**) of the fungal community and the Sobs index (**B**) and Shannon index (**D**) of the bacterial community at the OTU level. The boxes represent the average values of three biological replicates for each treatment, and the significance was tested using a *t*-test. Different lowercase letters indicate significant differences at the *p* < 0.05 level. The principal coordinate analysis plots show the changes in the composition of fungal (**E**) and bacterial (**F**) communities in two plant niches (roots and rhizosphere soil) under four treatments. The two-dimensional principal coordinates based on the Bray–Curtis distance were analyzed by the Adonis method for multivariate permutation to test the microbial differences. Each point represents a single composition sample, and different colors in the figure represent different strain treatments and different niches. RDK and SDK represent plant roots and rhizosphere soil after treatment with the DK strain; RF18-3 and SF18-3 represent plant roots and rhizosphere soil after treatment with the F18-3 strain; RB0 and SB0 represent plant roots and rhizosphere soil after treatment with the B0 strain; and RCK and SCK represent the plant roots and rhizosphere soil of the control group.

The microbial α-diversity of all plant niches under different treatments was calculated. The fungal community was more sensitive to strain treatments than the bacterial community, and significant differences could be found in the effects of DK, F18-3, and B0 treatments on the diversity of endophytic and rhizosphere fungi (Figure 4A–D). Specifically, DK and F18-3 treatments significantly reduced the richness (Sobs index: 21.77 ± 5.36%) and diversity (Shannon index: 20.69 ± 0.58%) of the endophytic fungal community, but the B0 treatment improved the diversity (Shannon index: 17.44%) but reduced the richness (Sobs index: 16.77%) of the endophytic fungal community. For bacterial α-diversity, the three strain treatments decreased the Shannon index of endophytic bacteria by 4.75 ± 3.24% but imposed little influence on richness.

For the α-diversity of rhizosphere soil fungal and bacterial communities, the DK and F18-3 treatments obviously improved the diversity of the fungal community (Shannon index: 12.97 ± 1.85%) and the richness of the bacterial community (Sobs index: 17.38 ± 0.28%) but had no significant effect on the richness of the fungal community and the diversity of the bacterial community. However, the B0 treatment significantly increased the diversity (Shannon index: 18.92%) and richness (Sobs index: 18.75%) of the fungal community, as well as the richness (Sobs index: 11.92%) of the bacterial community.

Further Kruskal–Wallis tests were conducted on the Shannon and Sobs indices of different strain treatments, and the results show that the *p* values for endophytic fungi, rhizosphere fungi, endophytic bacteria, and rhizosphere bacteria were (0.025, 0.025), (0.019, 0.023), (0.228, 0.154), and (0.622, 0.053), respectively (Table S7). These results further indicate that different strain treatments had significant effects on the α-diversity of endophytic and rhizosphere fungi.

At the genus level, PCoA based on the Bray–Curtis distance was employed to assess the differences in microbial communities among plant niches of various strain treatments to detect the reliability of the data and the treatment effects. It is clear that different strain treatments affected the community structures of fungi (Adonis: R^2^ = 0.922, *p* = 0.001; Anosim: R^2^ = 0.9448, *p* = 0.001) and bacteria (Adonis: R^2^ = 0.886, *p* = 0.001; Anosim: R^2^ = 0.777, *p* = 0.001). On the other hand, the impact of various strain treatments on fungal β-diversity was more obvious than that on bacterial β-diversity (the size of R^2^) (Figure 4E,F).

### 3.4. Microbial Groups Potentially Related to Strain Regulation

To further investigate the fungal and bacterial taxa that may respond to strain treatment and participate in regulating plant growth, the LEfSe algorithm was applied, and a total of 111 significantly responsive taxonomic units (from phylum to species, log10 (LDA) score > 4 and *p* < 0.05) were identified. The number of biomarkers increased in three strain treatments. Among them, the increase in the local fungus treatment (DK and F18-3) reached 47.62%, which is higher than that in the B0 treatment.

**Figure 5:**
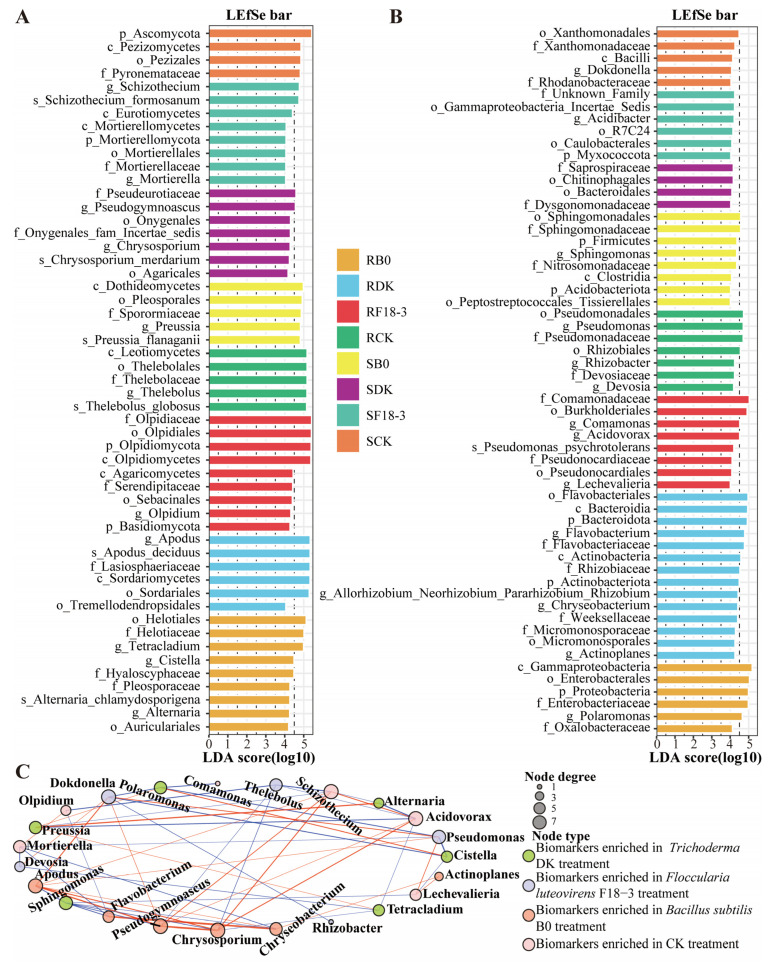
Biomarker identification and interaction analysis. The LEfSe (LDA effect size) discrimination result graph is used to display the endophytic and rhizosphere fungal (**A**) and bacterial (**B**) community biomarkers with significant differences among the four treatments. The length of the LEfSe bar represents the influence size of the biomarker. The LDA score threshold is log10 (LDA score) > 4, with *p* < 0.05. The higher the LDA score, the greater the influence of species abundance on the difference effect. The interaction network of plant root and rhizosphere biomarkers after DK, F18-3; (**C**) B0 and CK treatments is shown (|r| > 0.7, and *p* < 0.05). The color of each node represents the biomarkers of different ecological niches of fungi and bacteria, and the size of the node represents the number of biomarkers significantly related to it. The larger the node, the more biomarkers it is related to. The red lines represent positive correlations, and the blue lines represent negative correlations.

Twenty-four plant–endophyte fungi and twenty rhizosphere fungi were identified as the biomarkers after strain treatments (Figure 5A). Specifically, it was observed that after the DK treatment, fungi including *Apodus*, *Apodus deciduus*, *Chrysosporium*, *Chrysosporium merdarium*, and *Pseudogymnoascus* were enriched in the endophytes or rhizosphere of plants. After the F18-3 treatment, *Olpidium*, *Schizothecium*, *Schizothecium formosanum*, and *Mortierella* were enriched in the endophytes or rhizosphere of plants. Nevertheless, after the B0 treatment, endophytic *Cistella*, *Tetracladium*, *Alternaria*, and *Alternaria chlamydosporigena*, and rhizosphere *Preussia* and *Preussia flanaganii* were significantly enriched in plants. In addition, endophytic fungi *Thelebolus* and *Thelebolus globosus* were improved in the plants of the CK group.

After strain treatments, twenty-eight endophytic bacteria and eighteen rhizosphere bacteria were identified as the biomarkers of plants (Figure 5B). After the DK treatment, endophytic bacteria *Flavobacterium*, *Allorhizobium*-*Neorhizobium*-*Pararhizobium*-*Rhizobium*, *Actinoplanes*, and *Chryseobacterium* were clearly enriched in plants. Endophytic bacteria *Lechevalieria*, and *Comamonas* and rhizosphere bacterium *Acidibacter* were significantly enriched in plants after the F18-3 treatment. After the B0 treatment, *Polaromonas* and *Sphingomonas* were significantly enriched in plants. However, *Pseudomonas*, *Rhizobacter*, *Devosia*, and *Dokdonella* were significantly enriched in plants of the CK group.

To further investigate the correlations among these potential biomarkers and their competitive or cooperative relationships, co-expression networks were constructed for the biomarkers in different strain treatments. Interestingly, the biomarkers enriched in *Trichoderma* DK, including *Apodus*, *Chrysosporium*, and *Pseudogymnoascus*, have potential cooperative relationships with more biomarkers and exhibit significant positive correlations with each other. However, these biomarkers have no direct association with plant pathogenic fungi. The biomarker of *Acidovorax* enriched in *Floccularia luteovirens* F18-3 is negatively correlated with *Alternaria* and *Preussia*, while both *Acidovorax* and *Schizothecium* are positively correlated with *Pseudogymnoascus*. Additionally, *Preussia* and *Alternaria* enriched in B0 are positively correlated, *Polaromonas* and *Cistella* are also positively correlated, and there is a potential cooperative relationship between *Preussia* and *Polaromonas* (Figure 5C).

Furthermore, the functional characteristics related to strain treatment were predicted through FUNGuild and FAPROTAX analysis according to the identified biomarkers. It was found that undefined saprotroph was the most common functional group of fungi, with the highest relative abundance of 59.39% in the plant root treated with DK, and the relative abundance in the rhizosphere soil of plants after strain treatment all increased (by 79.74 ± 15.52%) (Figure S5A). Interestingly, local fungal treatments enhanced the plant saprotroph–wood saprotroph ecological functional group in the rhizosphere soil (by 96.73 ± 54.07%). Moreover, the B0 treatment strengthened the ectomycorrhizal–fungal parasite–plant pathogen–wood saprotroph (by 24.15%) and the animal pathogen–endophyte–plant pathogen–wood saprotroph (by 4.77%) in the plant root system. The dominant ecological functional group in the CK group was dung saprotroph, with relative abundances of 36.21% in the roots and 33.04% in the rhizosphere soil (Figure S5A). However, the strain treatments imposed little impact on the functional groups of bacteria in plants and rhizosphere soil, with chemoheterotrophy and aerobic chemoheterotrophy as the main groups. Notably, the B0 treatment significantly reduced the relative abundance of these two functional groups in the plant roots (Figure S5B).

### 3.5. Influence of Strain Treatment on Metabolism of Plant Rhizosphere Soil

**Figure 6:**
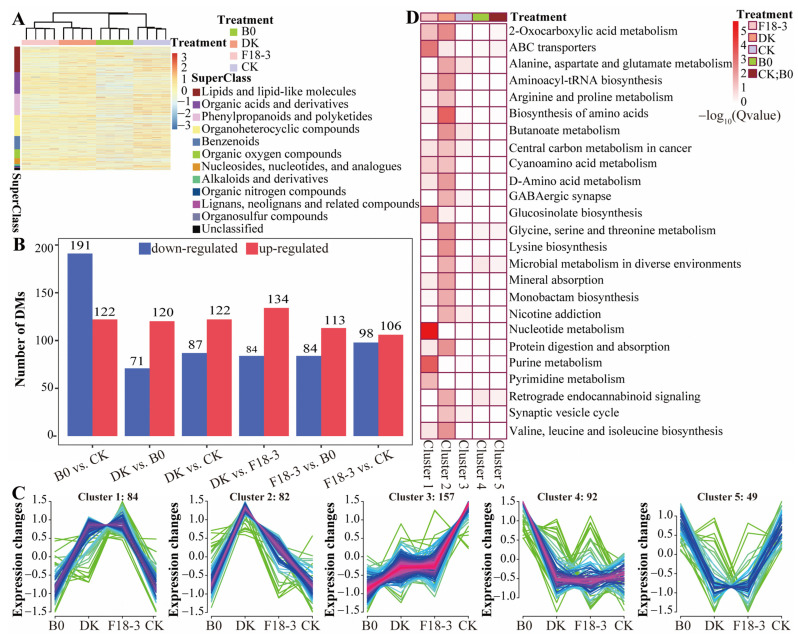
The effects of strain treatments on the accumulation of metabolites in the rhizosphere soil of plants. (**A**) A cluster heatmap of metabolites in the rhizosphere soil of plants after four treatments. Different colors in the row annotation column represent different supercategories, and different colors in the column annotation column represent different strain treatments. (**B**) The number of differential metabolites (DMs) identified in different comparison groups based on VIP of OPLS-DA and univariate statistical analysis (VIP > 1, and *p* < 0.05). (**C**) K-means clustering analysis of 464 DMs. The x-axis represents four different treatments, and the y-axis depicts the relative changes in the metabolite content. (**D**) KEGG enrichment analysis of DMs in five clusters. The depth of color is determined by −log10 (Qvalue), with darker colors indicating smaller Qvalues and more significant metabolic and biosynthetic pathways. The annotation column shows the treatment group corresponding to each cluster.

A metabolomic analysis of rhizosphere soil from different treatments of plants was conducted using LC-MS/MS. After quality control, 2516 metabolites were found, among which 1216 were annotated to the HMDB database and 526 to the KEGG database. The identified metabolites in all samples involved 255 lipids and lipid-like molecules, 216 organic acids and derivatives, 213 phenylpropanoids and polyketides, and other compounds (Table S8). As shown in the clustering heatmap (Figure 6A), microbial treatments obviously changed the metabolic profiles of plant rhizosphere soil, and the metabolic profiles of the DK and F18-3 treatments were significantly different from those of the B0 treatment.

A total of 464 metabolites with differential accumulation between different comparison groups (VIP ≥ 1 and *p* < 0.05) were obtained, and more clearly up-regulated DMs were obtained using the fungal treatment (Figure 6B, Table S9). Additionally, K-means clustering analysis showed that five different clusters (cluster1–cluster5) corresponded to four different treatments: DK (cluster2), B0 (cluster4 and cluster5), F18-3 (cluster1) and CK (cluster3 and cluster5), further indicating that the rhizosphere metabolite accumulation patterns of the DK and F18-3 treatments were different from those of the B0 treatment (Figure 6C). KEGG analysis of metabolites revealed that metabolites in different clusters were involved in multiple metabolic and biosynthetic pathways (Figure 6D). Specifically, the DK group was significantly enriched in the most pathways, totaling 20, among which the top 5 were biosynthesis of amino acids, 2-oxocarboxylic acid metabolism, lysine biosynthesis, aminoacyl-tRNA biosynthesis, and butanoate metabolism; the F18-3 group was significantly enriched in 6 pathways, including nucleotide metabolism, purine metabolism, ABC transporters, glucosinolate biosynthesis, pyrimidine metabolism, and 2-oxocarboxylic acid metabolism. Among these pathways, the 2-oxocarboxylic acid metabolism pathway was significantly enriched in both the DK and F18-3 treatment groups, but no significantly enriched metabolic and biosynthetic pathways were detected in the B0 treatment and CK group. Therefore, DK had the most significant impact on the rhizosphere metabolism of plants, followed by F18-3, while B0 had the smallest effect.

### 3.6. Rhizosphere Metabolites Drive the Assembly of Microorganisms Associated with Plant Growth

**Figure 7:**
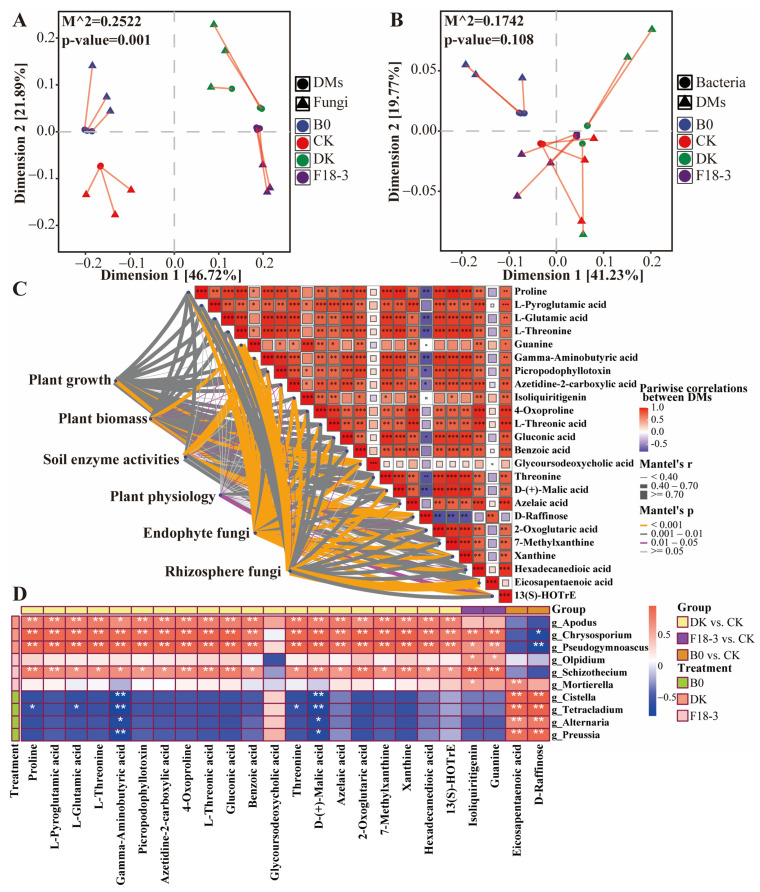
Correlation analysis between biomarkers and differential metabolites. Procrustes analysis between the metabolome and fungal (**A**) or bacterial (**B**) communities. The microbial communities consist of plant roots and rhizosphere fungi or bacteria. PCoA was used to reduce the dimensionality of the microbiome and metabolome data, followed by Procrustes analysis. M^2^ represents the sum of squared distances between matching sample pairs, and a smaller value indicates a stronger correlation between the two datasets. *p* < 0.01 indicates a highly consistent change between the two datasets; *p* < 0.05 indicates a consistent change; *p* > 0.05 indicates an insignificant association trend. The lines represent the Procrustes residuals of the two ordered configurations, and shorter lines indicate higher consistency between the two datasets. (**C**) The Mantel test was used to analyze the relationship between plant variables and fungal biomarkers (determined by the Bray–Curtis distance) and DMs (determined by the Euclidean distance). The Mantel’s r value is represented by the edge width, while the statistical significance is indicated by the edge color. The pairwise correlations between DMs are represented by a color gradient reflecting Spearman’s correlation coefficient. Plant growth (shoot length, root length, and stem diameter), plant biomass (shoot biomass and root biomass), soil enzyme activities (S-AL, S-GC, S-PPO, and S-CL), plant physiology (total protein, PRO, SSu, and chlorophyll content), endophyte fungi (26 biomarkers in plant roots) and rhizosphere fungi (24 biomarkers in plant rhizosphere). (**D**) A heatmap based on the Pearson correlation indicates the association between core metabolites and fungi (biomarker genera). Metabolites are at the bottom, and fungi are on the right. The stars indicate Pearson correlation coefficients (** *p* < 0.01; * 0.01 < *p* < 0.05), and the “blue to red” color gradient represents the Pearson correlation coefficient values, with deeper red indicating a larger positive Pearson correlation coefficient and deeper blue indicating a larger negative Pearson correlation coefficient. The column annotation bars in different colors represent DMs from different comparison groups, while the row annotation bars in different colors represent fungi from different treatment groups.

To investigate the relationship between rhizosphere metabolites and plant-growth-related microorganisms, 464 identified DMs and 111 potential biomarkers related to strain regulation were selected for Procrustes analysis. First, PCoA was used to reduce the dimensions and sort the DMs and biomarkers, followed by Procrustes analysis to compare the similarities and variations between DMs and biomarkers. Fungal biomarkers significantly corresponded to metabolite synthesis (M^2^ = 0.2522, *p* = 0.001), but bacterial biomarkers were not (M^2^ = 0.1742, *p* = 0.108) (Figure 7A,B).

To identify key metabolites that regulate plant growth and drive the assembly of beneficial microorganisms, a metabolome–microbiome interaction analysis was conducted on fungal biomarkers and DMs. The DMs (log2 (FC) > 0 and VIP > 1.5) corresponding to the comparison groups of DK vs. CK, F18-3 vs. CK, and B0 vs. CK in cluster2, cluster1, and cluster4 were further screened out, ultimately resulting in 24 DMs. Except for isoliquiritigenin and guanine in the F18-3 vs. CK comparison group and eicosapentaenoic acid and D-Raffinose in the B0 vs. CK comparison group, the remaining 20 DMs all originated from the DK vs. CK comparison group (Table S10). A total of 24 DMs and 50 plant root and rhizosphere biomarker fungi (except for the pathogenic fungi *Cistella*, *Alternaria*, and *Alternaria chlamydosporigena*), as well as the indicators related to plant growth, biomass accumulation, plant physiology and rhizosphere soil enzyme activity, were used for the Mantel test. Most of the selected DMs were significantly correlated with plant growth, biomass accumulation, plant physiology, rhizosphere soil, and plant root and rhizosphere biomarker fungi (Figure 7C, Table S11). D-(+)-malic acid, gamma-Aminobutyric acid (GABA), and glycoursodeoxycholic acid were identified as the primary predictors. Specifically, 20 DMs from the DK vs. CK comparison group were significantly correlated with plant growth, while the 4 DMs from the F18-3 vs. CK and B0 vs. CK comparison groups were not significantly correlated. Plant biomass was most significantly correlated with isoliquiritigenin, glycoursodeoxycholic acid, and guanine (arranged in descending order of Mantel’s r), while plant physiology was most significantly associated with glycoursodeoxycholic acid, eicosapentaenoic acid, and 13(S)-HOTrE. In the rhizosphere soil of plants, enzyme activity exhibited significant correlations with D-Raffinose, eicosapentaenoic acid, and GABA. Additionally, isoliquiritigenin, glycoursodeoxycholic acid, and guanine were the core DMs driving the assembly of fungal biomarkers in plant roots, while eicosapentaenoic acid, guanine, and D-Raffinose were the core DMs driving the fungal biomarkers in rhizosphere soil.

In summary, DK treatment can drive the assembly of beneficial microorganisms by positively regulating the accumulation of more metabolites and can directly positively affect plant growth and the accumulation of beneficial biochemical substances in leaves through metabolites. However, F18-3 treatment had a more significant impact on the plant endophytic fungal community and plant biomass accumulation.

Finally, a correlation analysis between 24 DMs and 10 fungal biomarkers (genera level) was conducted (Figure 7D). The fungal biomarkers (*Apodus*, *Chrysosporium*, and *Pseudogymnoascus*) and key microbial group *Pseudeurotiaceae* enriched in the DK treatment group were all significantly positively corresponded to 20 DMs from the DK vs. CK comparison group. Additionally, D-(+)-malic acid and gamma-Aminobutyric acid were significantly negatively correlated with pathogenic fungi *Cistella* and *Alternaria*. However, eicosapentaenoic acid and D-Raffinose from the B0 vs. CK comparison group were significantly positively correlated with *Cistella* and *Alternaria*.

## 4. Discussion

In this work, the regulatory mechanisms of local fungi from the Qinghai–Tibet Plateau (*Trichoderma* DK and *Floccularia luteovirens* F18-3) and commercially available bacteria (*Bacillus subtilis* B0) affecting forage growth and rhizosphere microecology were systematically evaluated. The ecological restoration advantages of local fungi in extreme conditions and the driving mechanism of metabolic–microbial interaction were revealed.

### 4.1. Metabolites Regulate Plant Growth and Stress Resistance

Compared with the B0 strain, the DK and F18-3 strains improved shoot and root biomass by 23.43% and 195.58%, respectively, as well as soil nutrients (e.g., SOC and TN). This phenomenon may be caused by the unique physiological functions by the local fungi from the Qinghai–Tibet Plateau to adapt to the very cold, low-oxygen, and strong radiation environment over a long period of time. The DK and F18-3 treatments significantly increased the activity of S-AL and S-CL in the rhizosphere soil. S-AL and S-CL increase available carbon and nitrogen in the soil. More available carbon promotes rhizosphere microbial symbiosis and helps plants absorb phosphorus. Meanwhile, the increase in available nitrogen promotes plant protein synthesis and plant growth [51,52]. This may be one of the reasons why plants in the local fungal treatment group had more nutrients in the rhizosphere soil.

According to the metabolomic analysis, DK treatment significantly altered the metabolic profile of the plant rhizosphere, suggesting that DK strains affected plant growth by regulating a wide range of metabolites. The key metabolic pathways of DM enrichment were focused on biosynthesis of amino acids, 2-oxocarboxylic acid metabolism, protein digestion and absorption, and mineral absorption. This suggests that regulating the organic acid and nitrogen metabolic pathways is a key strategy for DK treatment to enhance organic cycling. Amino acids are important components of soil organic nitrogen and an important source of nutrients for soil microorganisms, which can use amino acids as precursors in their metabolic processes to synthesize plant growth regulators through biological pathways, stimulating plant growth and regulating plant physiological processes. In addition, DK treatment activates lysine and aminoacyl-tRNA biosynthesis pathways, which may accelerate protein biosynthesis and is important for soil health. Soil proteins contribute to soil health by inducing soil aggregate formation and soil organic matter (SOM) stabilization [53]. DK treatment enhanced the microbial synergy in the rhizosphere and roots and promoted protein absorption in plants. This phenomenon aligns with the obvious increase in the total protein content in the leaves of forage in the DK treatment group; proteins are essential substances for plants and act a key part in the formation and metabolism of plant cells and tissues.

Among the over 2000 metabolites identified, organic acids and their derivatives, lipids, and lipid-like molecules are the most abundant. Organic acids, as carbon sources and chelating agents, support microbial growth and directly participate in nutrient activation. For instance, gluconic acid can bind with metal ions such as Fe^3+^ and Al^3+^, releasing fixed phosphate [54]. DK treatment specifically accumulates secondary metabolites, including azelaic acid, malic acid, and 2-oxoglutaric acid, directly promoting the decomposition of organic matter and nutrient release. Plants and soil microorganisms can regulate the release of phosphorus and its hydrolysis by azelaic acid [55]. Plants and microorganisms can enhance the activation of compounds like phytate phosphorus by malic acid [56]. 2-Oxoglutaric acid significantly improves soil fertility. It can promote the transformation of nitrogen in the soil, enhance the oxidation of ammonium nitrogen by nitrifying bacteria, and make nitrogen available to plants in the form of nitrate nitrogen, thereby increasing soil nitrogen availability [57]. Notably, compared with F18-3, the DK treatment did not significantly change the TP content but significantly reduced the TN, AN, and AP contents in the rhizosphere soil of plants. In this sense, DK treatment regulates the release of N and P through rhizosphere metabolites. In addition, it promotes the absorption of soil nutrients by plants, thus maintaining plant growth. This may stem from the fact that the plant growth effect was the best after the DK treatment, while the nutrient content in the rhizosphere soil was generally lower than that of the F18-3 treatment and the control group.

Some secondary metabolites play special beneficial roles in plant growth and resistance to adverse environments. DK treatment induces the high accumulation of metabolites such as azelaic acid, GABA, threonine, and malic acid. In *Arabidopsis thaliana*, azelaic acid has been found to induce systemic acquired resistance (SAR) and act as a mobile signal for SAR [58]. Additionally, azelaic acid can trigger SAR against plant pathogens [59]. In fact, azelaic acid can effectively promote seed germination and the growth of the hypocotyl and roots of plant seedlings [60]. Therefore, the DK treatment showed the best plant growth effect and significantly reduced MDA. GABA functions as a defense substance and signal molecule in various physiological processes, helping plants cope with biotic and abiotic stresses; additionally, the synergistic effect of GABA and gibberellin can improve plant adaptability and promote plant growth [61,62]. Threonine and L-Threonine are important amino acids that constitute proteins and are significant components of soil nitrogen. Furthermore, most plants can directly absorb amino acids (an essential part in plant stress resistance) [63]. Threonine enhances plant stress resistance by participating in the regulation of the antioxidant system, like the synthesis of glutathione [64]. Amino acids (such as proline and valine) act as osmotic regulators and may regulate microbial behavior through quorum sensing [65]. For instance, valine treatment can activate rice resistance to rice blast [66,67]. This indicates that the DK treatment alleviates oxidative stress and enhances plant cold resistance through metabolites.

### 4.2. Metabolite–Microbe Synergy Drives Plant Growth

Root exudates and microbial metabolites play a crucial role in plant growth, adaptation to environmental changes, and the formation of plant–microbe and microbe–microbe relationships. However, research on the correlation between microbial communities and metabolomes remains scarce. Specific metabolites can attract beneficial microbes to promote plant growth, while others can act as antibacterial substances to inhibit pathogen growth [68,69]. The C compounds of low molecular weight in root exudates, including sugars, organic acids, and amino acids, are easily assimilated by microbes and play a major role in regulating the dynamics of the rhizosphere microbial community [70]. The Procrustes analysis demonstrated that the fungal community was significantly correlated with the synthesis of metabolites. This suggests that the alteration in metabolites in the rhizosphere soil of plants is an important driving force for the reorganization of the microbial community.

DK treatment increased the secretion of lipids, polysaccharides, and organic acids. Lipids play a vital role in microbe–plant recognition, and lipids may enhance plant stress resistance through membrane receptor-mediated immune responses [71]. Organic acids, as a carbon source, can attract phosphorus-solubilizing bacteria (e.g., *Bacillus* and *Cellvibrio*), as well as nitrogen-fixing bacteria (such as *Azospirillum*), forming a “phosphorus-solubilizing, nitrogen-fixing” functional consortium [72]. Some genera responded strongly to the DK treatment, and DK treatment could recruit certain beneficial microbes to promote plant growth. Biomarkers, including *Pseudogymnoascus*, *Apodus deciduus*, *Chryseobacterium*, *Flavobacterium*, and *Actinoplanes*, were significantly enriched in the DK treatment group. The high abundance of *Pseudogymnoascus* in the rhizosphere helps with nutrient cycling, enabling plants to better adapt to the environment and exerting antagonistic effects against pathogens [73]. *Apodus deciduus* is a key saprotrophic fungus that promotes the decomposition of rhizosphere organic matter [74]. Members of the *Chryseobacterium* genus can act as beneficial bacteria for plant growth promotion and biological control [75]. *Flavobacterium* can produce auxins and siderophores and promotes plant growth [76]. *Actinoplanes* spp. can enhance plant antioxidant and detoxification defense mechanisms, thereby promoting plant growth, and they are important antibiotic producers, potentially inhibiting pathogen growth [77]. Notably, this work demonstrated a significant correlation between many significantly enriched metabolites and beneficial microbes in the DK treatment group.

DK treatment specifically accumulated key metabolites like azelaic acid, benzoic acid (BA), and malic acid and activated microbial metabolism in diverse environments (ko01120), promoting the colonization of beneficial microorganisms (such as *Pseudogymnoascus*, *Chryseobacterium*, *Flavobacterium*, etc.). Meanwhile, D-(+)-malic acid and GABA inhibit pathogenic fungi *Cistella* (−84.20%) and *Alternaria* (−58.53%). Studies have shown that adding BA to the soil can increase the relative abundance of nitrogen-fixing microorganisms in the soil of gramineae plants, increase the copy number of *NifH* genes in the soil, and thereby enhance the nitrogen content and nitrogen-fixing ability of plants [78]. Meanwhile, BA is one of the compounds with significant antibacterial activity [79]. Malic acid can recruit rhizosphere-promoting bacteria, such as *Bacillus subtilis* and *Pseudonocardia*, and *Pseudonocardia* has potential antibacterial functions [80,81]. Azelaic acid affects microbial diversity in the soil and regulates the bacterial community by promoting the growth of symbionts [59]. The results show that nitrogen-fixing bacteria *Allorhizobium*-*Neorhizobium*-*Pararhizobium*-*Rhizobium* are significantly enriched with DK treatment. Hence, the DK strain can be adopted to promote the diversity of rhizosphere microorganisms and inhibit the growth of plant pathogenic fungi. These positive effects lead to improved plant adaptability in high-altitude conditions.

### 4.3. Synergistic Interactions Among Microorganisms Drive Plant Growth

In addition to analyzing the changes in microbial groups and rhizosphere metabolites, exploring the changes in microbial interactions through microbial network can provide clues to the impact of DK-strain-induced microbial group changes on plant growth. The DK treatment enhanced the complexity of the microbial interaction network, with *Pseudogymnoascus* as a hub, promoting the proliferation of saprotrophs (undefined saprotroph) and accelerating the cycling of organic matter. In contrast, the B0 treatment enriched potential pathogenic fungi (*Cistella* and *Alternaria*). Previous studies have shown that complex interactions are more conducive to resisting environmental disturbances [82]. Thus, the higher complexity of synergistic interactions may indicate that DK strain treatment causes plant roots and rhizosphere microorganisms to have greater resilience to high-altitude environments. The synergistic interactions among microorganisms make plants more conducive to their growth. *Rhizobia* and PGPR (plant-growth-promoting rhizobacteria) synergistically promote plant growth through mechanisms like nutritional complementarity and plant growth promotion synergy [83]. The PGPR-produced plant hormones promote the formation of root nodules, while *Rhizobia* provide nitrogen for plant growth through nitrogen fixation, promoting plant growth and thereby expanding the growth space of PGPR [84]. Additionally, the nitrogen fixed by *Rhizobia* provides a nitrogen source for PGPR growth, and the small molecules released during the growth of PGPR can provide nutrients needed for *Rhizobia* growth [85]. Notably, the genus *Trichoderma*, a filamentous fungus, is a widely studied microorganism with antagonistic effects against plant pathogens. *Trichoderma* grows towards fungal pathogens and releases toxic compounds and a series of lyases, such as chitinase, glucanase, and protease. These enzymes promote *Trichoderma* penetration into the host and utilization of nutrients provided by the host [86]. Although fungal treatment reduces fungal α-diversity (the Shannon index decreases by 3.91% to 32.82%), it specifically enriches multiple beneficial functional microbial groups, forming a microecology adapted to high-altitude environments. Although B0 treatment increases bacterial richness, it enriches multiple pathogenic fungi and reduces microbial interactions, indicating that commercialized microbial agents may have weakened functions in high-altitude environments.

In conclusion, the DK strain shows the best effect on plant growth and stress resistance at 4126 m on the Qinghai–Tibet Plateau, outperforming another local fungi F18-3 and the commercially available bacteria B0. DK treatment drives microbial assembly and is beneficial to microbial aggregation by regulating the accumulation of more metabolites and metabolic and biosynthetic pathways. In addition, it promotes the synergy between plant roots and rhizosphere microorganisms. This characteristic can be used for vegetation restoration in high-altitude areas to enhance the effectiveness of microbial remediation processes.

The selected strains DK, F18-3, and B0 in this study, although belonging to different genera, can all be classified as plant-growth-promoting microorganisms, involving nutrient transformation (such as phosphorus solubilization and nitrogen fixation), stress alleviation, and improvement of the rhizosphere microenvironment. This functional commonality provides a basis for cross-taxonomic comparison. In this study, *Bacillus subtilis* B0 was chosen as the control strain, not only to compare the differences between fungi and bacteria but because it is the most widely used commercial strain in agriculture and ecological restoration, and its effectiveness in temperate regions has been confirmed. The results of this study show that although B0 can slightly promote the growth of forage, it significantly reduces the root biomass of forage (by 11.02%) and enriches plant pathogenic fungi (*Cistella* and *Alternaria*), revealing its limitations in extreme environments. It must be admitted that cross-taxonomic comparison cannot completely eliminate the influence of species differences on the experimental results. For example, the specific promotion of underground biomass by F18-3 (by 162.99%) may be related to its characteristics as a mycorrhizal fungus rather than simply a “local advantage”. Future studies can further introduce “same species but different locations strains”, and by comparing their plant-growth-promoting effects in high-altitude mining areas, the plant-growth-promoting mechanism of local fungi can be more directly analyzed. The current work provides a “local strain–metabolic regulation–microbial network” technical paradigm for ecological restoration in high-altitude extreme environments, but the long-term ecological effects and cross-regional applicability still need further verification.

## 5. Conclusions

Local fungi play a key role in promoting the growth of plants in extremely high-altitude mining areas on the Qinghai–Tibet Plateau. This study systematically analyzed the effects of local fungi from the Qinghai–Tibet Plateau (DK and F18-3) and a commercially available bacterium (B0) on the growth of forage and the rhizosphere microecology and reached the following conclusions: Local fungi can significantly promote the growth and biomass accumulation of plants in extremely high-altitude areas of the Qinghai–Tibet Plateau and improve the biological characteristics and microbial community structure of rhizosphere soil. Importantly, DK significantly increases fungal diversity and the amount of unique bacterial genera in the rhizosphere soil of plants but also aggregates the most biomarkers and beneficial microorganisms. The DK treatment induces the accumulation of twenty highly different metabolites (DMs) in rhizosphere soil, which are significantly positively correlated with plant growth and biomass. Among them, D-(+)-malic acid and GABA can inhibit pathogenic fungi *Cistella* and *Alternaria*. The local fungus DK mainly inhibits the growth of pathogenic fungi by regulating the composition of metabolites in the plant rhizosphere, while the strain F18-3 exerts its antibacterial effect by enriching specific beneficial rhizosphere microorganisms. Further long-term monitoring and experimental studies are needed to elucidate the mechanisms of indigenous fungi in regulating inter-root metabolites and driving beneficial microbial aggregation.

## Data Availability

The raw sequence data was deposited in the Sequence Read Archive (SRA) of the National Center for Biotechnology Information (NCBI) database (bacterial 16S rRNA gene: accession no. PRJNA1187823; fungal ITS: accession no. PRJNA1187810).

## Supplementary Materials

The following supporting information can be downloaded at https://www.mdpi.com/article/10.3390/microorganisms13081752/s1, Figure S1: Location of the study area and study design. B0: *Bacillus subtilis* B0 mixed with sheep board manure; Figure S2: Effects of strain treatment on physical and chemical properties and enzyme activities of plant rhizosphere soil; Figure S3: Saturation dilution curves and rank abundance curves of fungal and bacterial communities; Figure S4: The number of shared/unique fungal (A) and bacterial (B) OTUs; Figure S5: Functional analysis of biomarker microbial groups identified in plant roots and rhizosphere soil after strain treatment; Table S1: Information of tested plant growth promoting microorganisms; Table S2: Information of tested forage seed; Table S3: Soil nutrient content before experiment; Table S4: Taxonomic classification of fungal OTUs; Table S5: Taxonomic classification of bacterial OTUs; Table S6: Bacterial and fungal communities in plant microbial niches. Bacterial microbiota on genera level; Table S7: The Kruskal-Wallis H test on α-diversity of different strain treatments; Table S8: Classification of identified metabolites in rhizosphere soil samples; Table S9: Total number of accumulated differential metabolites (DMs) across different comparison groups; Table S10: List of 24 key accumulated differential metabolites (DMs) screened from DK vs. CK, F18-3 vs. CK, and B0 vs. CK comparison groups; Table S11: Mantel test results.
