# Supplementary material for: Local Fungi Promote Plant Growth by Positively Affecting Rhizosphere Metabolites to Drive Beneficial Microbial Assembly
Source: Microorganisms. 2025 Jul 26;13(8):1752. doi: 10.3390/microorganisms13081752 (PMC12388802; doi:10.3390/microorganisms13081752)
Supplement: Supplementary file 1 [file microorganisms-13-01752-s001.zip › Supplementary Figures.pdf]

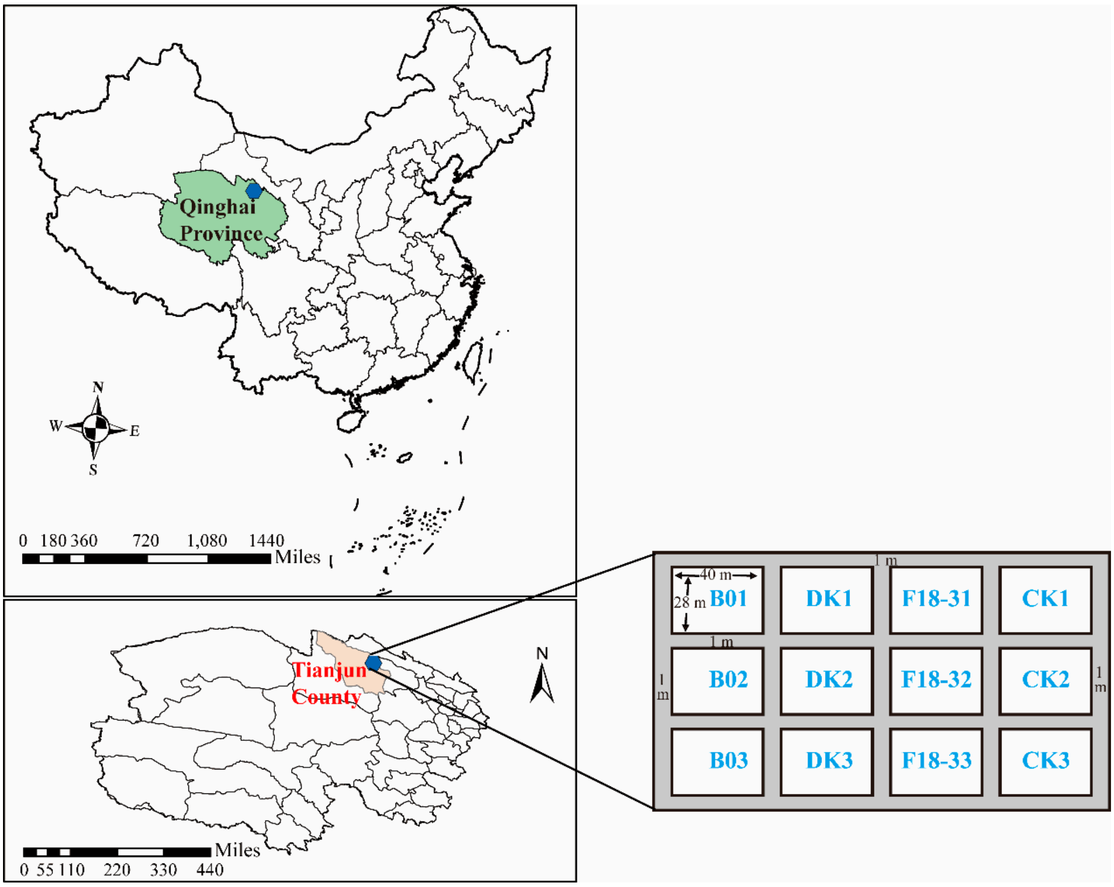

Figure S1. Location of the study area and study design. B0: *Bacillus subtilis* B0 mixed with sheep board manure; DK: *Trichoderma* sp. DK mixed with sheep board manure; F18-3: *Floccularia luteovirens* F18-3 mixed with sheep stool; CK: blank control.

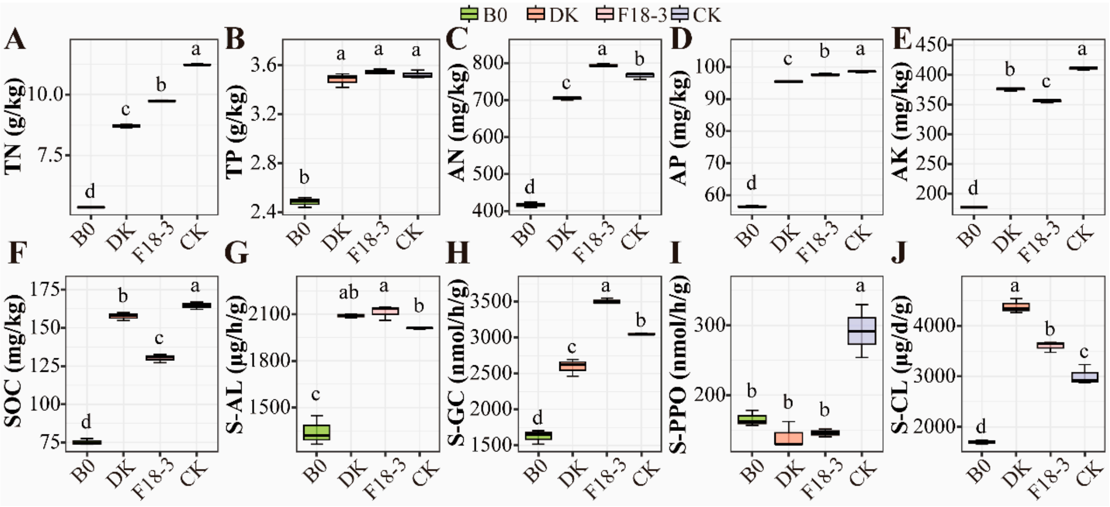

Figure S2. Effects of strain treatment on physical and chemical properties and enzyme activities of plant rhizosphere soil. (A) TN; (B) TP; (C) AN; (D) AP; (E) AK; (F) SOC; (G) S-AL; (H) S-GC; (I) S-PPO; (J) S-CL. Different lowercase letters above the column represent significant differences ($p < 0.05$), n=3.

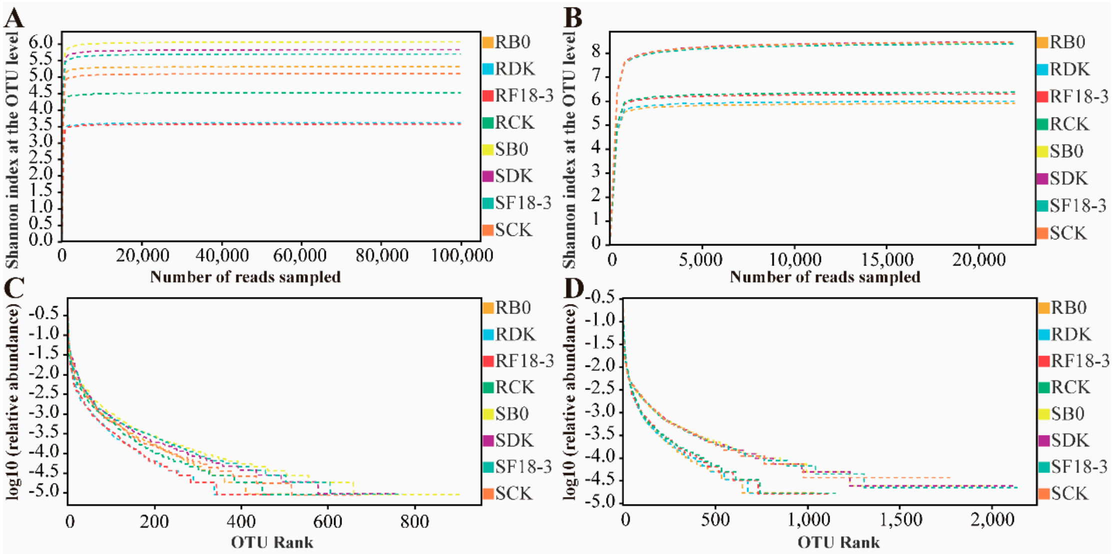

Figure S3. Saturation dilution curves and rank abundance curves of fungal and bacterial communities. Shannon dilution curves of fungal (A) and bacterial (B) community composition at the OTU level. The dilution curve is calculated by using the relative proportion of known OTUs in the measured sequences to obtain the expected value of the Alpha diversity index when n tags (n is less than the total number of measured tag sequences) are extracted, and then a curve is drawn based on a set of n values (generally an arithmetic sequence less than the total number of sequences) and their corresponding expected values of the Alpha diversity index. The x-axis represents the amount of randomly sampled sequencing data, and the y-axis represents the diversity index (Shannon index) at the OTU level. Rank abundance analysis of fungal (C) and bacterial (D) community groups at the OTU level. The OTUs in the samples are sorted by relative abundance from large to small to obtain the corresponding number, and then the sorted number of OTUs is taken as the x-axis and the relative abundance of OTUs as the y-axis, and these points are connected by a line. RDK and SDK represent the plant roots and rhizosphere soil treated with the DK strain; RF18-3 and SF18-3 represent the plant roots and rhizosphere soil treated with the F18-3 strain; RB0 and SB0 represent the plant roots and rhizosphere soil treated with the B0 strain; RCK and SCK represent the plant roots and rhizosphere soil of the control group.

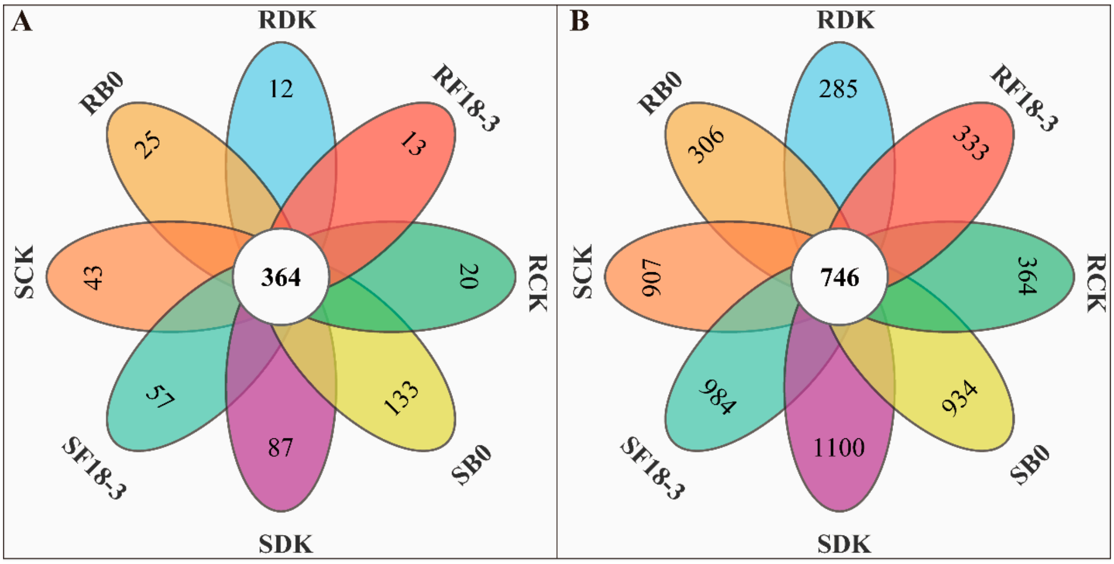

Figure S4. The number of shared/unique fungal (A) and bacterial (B) OTUs. RDK and SDK represent the plant roots and rhizosphere soil treated with DK strain, respectively; RF18-3 and SF18-3 represent the plant roots and rhizosphere soil treated with F18-3 strain, respectively; RB0 and SB0 represent the plant roots and rhizosphere soil treated with B0 strain, respectively; RCK and SCK represent the plant roots and rhizosphere soil of the control group, respectively.

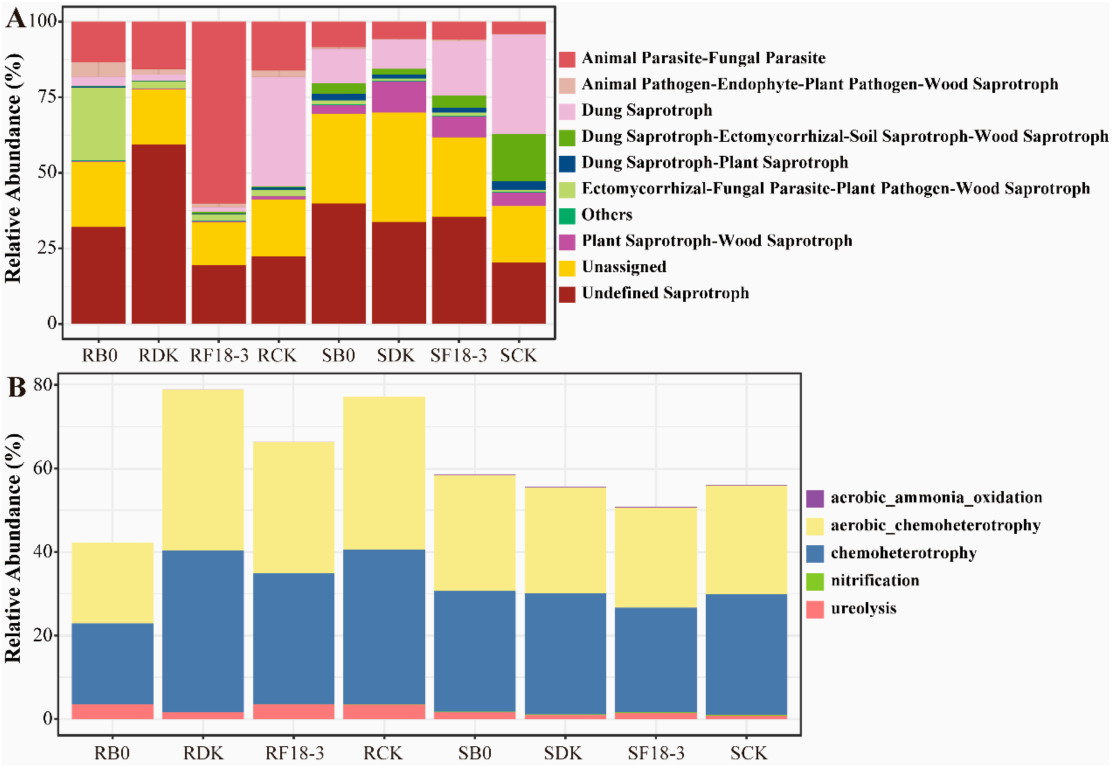

Figure S5. Functional analysis of biomarker microbial groups identified in plant roots and rhizosphere soil after strain treatment. A: Changes in the composition of predicted fungal functional guilds (top 9) by FUNGuild analysis; B: Predicted bacterial functional spectra by FAPROTAX analysis. RDK and SDK represent plant roots and rhizosphere soil after treatment with DK strain; RF18-3 and SF18-3 represent plant roots and rhizosphere soil after treatment with F18-3 strain; RB0 and SB0 represent plant roots and rhizosphere soil after treatment with B0 strain; RCK and SCK represent plant roots and rhizosphere soil of the control group.
